# Folic Acid–Functionalized MWCNT-Conjugated Zirconium Oxide Nanoparticles for Targeted Cancer Cell Delivery of Astaxanthin

**DOI:** 10.1155/bca/4077233

**Published:** 2025-10-12

**Authors:** Han-Sol You, Anbazhagan Sathiyaseelan, Myeong-Hyeon Wang, Jong-Suep Baek

**Affiliations:** ^1^Department of Bio-Health Convergence, Kangwon National University, Chuncheon 24341, Republic of Korea; ^2^BeNatureBioLab, Chuncheon 24206, Republic of Korea

**Keywords:** anticancer, astaxanthin, folic acid, multiwalled carbon nanotubes, targeting, zirconium oxide nanoparticles

## Abstract

In this study, zirconium oxide nanoparticles (ZrO_2_ NPs) were synthesized using astaxanthin (AST) rich extract (AZ) and subsequently conjugated with multiwalled carbon nanotubes (MWCNTs) (AZM) and functionalized with folic acid (FA) (FAZM) to develop a cancer-targeting nanocomposite with enhanced anticancer efficacy. The physicochemical properties of the synthesized materials were characterized using transmission electron microscopy (TEM), dynamic light scattering (DLS), electrophoretic light scattering (ELS), X-ray diffraction (XRD), and Fourier transform infrared spectroscopy (FT-IR). FAZM exhibited the highest antioxidant activity, with IC_50_ values of 822.78 μg/mL against ABTS and 320.70 μg/mL against DPPH free radicals. Biocompatibility assessments revealed that FAZM exhibited little cytotoxicity in normal human skin cells and demonstrated improved hemocompatibility, as confirmed by a hemolysis assay. Furthermore, FAZM significantly inhibited the proliferation of MDA-MB-231 breast cancer cells, inducing apoptosis and exhibiting potent cytotoxic effects (IC_50_: 115.84 μg/mL). These findings suggest that FA and MWCNTs enhance the cancer-targeting capability of AZ while maximizing its selective cytotoxicity against cancer cells. This study highlights that FA-functionalized MWCNT-conjugated ZrO_2_ NPs are a promising nanoplatform as an AST delivery system for targeted cancer therapy.

## 1. Introduction

Cancer remains one of the leading causes of mortality worldwide, with the World Health Organization (WHO) reporting that approximately 10 million people died from cancer in 2020. This number is projected to rise to around 12 million by 2030 [1]. Current cancer treatment methods include surgery, chemotherapy, and radiotherapy. While surgery is widely used for solid tumors, metastasis remains a significant challenge [2]. Chemotherapy, though effective, lacks specificity and affects both cancerous and normal cells, including bone marrow and immune cells, leading to severe side effects [3]. Similarly, radiotherapy, utilized in more than half of cancer patients, is highly toxic and can cause substantial side effects [4]. Therefore, there is an urgent need for targeted therapeutic strategies that selectively eliminate cancer cells while minimizing damage to normal tissues.

Metal oxide nanoparticles (NPs) can effectively promote redox reactions due to their high surface area and surface defects. Some metal oxide NPs can be activated in specific environments, such as acidic tumors (low pH) or increased hydrogen peroxide (H_2_O_2_) concentration, to exert targeted anticancer effects [5]. NPs-based drug delivery systems (DDSs) have emerged as promising approaches to enhance the efficacy of anticancer treatments while reducing systemic toxicity [6]. The use of NPs as drug carriers offers several advantages, including reduced drug dosage, improved biocompatibility, enhanced targeted drug delivery, and minimized toxicity [7]. Zirconium oxide NPs (ZrO_2_ NPs) have attracted attention as drug carriers due to their high surface area, biocompatibility, and controllable surface properties compared to other metal oxide NPs [8]. Their high surface area-to-volume ratio enables efficient drug loading, making them suitable candidates for nanomedicine applications [9].

Astaxanthin (AST), a naturally occurring carotenoid mainly produced by the marine microalgae *Haematococcus pluvialis* (*H. pluvialis*), has demonstrated potent antioxidant and anticancer properties [10]. AST has a polyene chain and an oxo ring structure with ketone and hydroxyl groups at both ends and has been reported to have superior antioxidant abilities than conventional antioxidants such as vitamin C and vitamin E [11]. As oxidative stress and reactive oxygen species (ROS) play key roles in cancer progression, AST's ability to modulate oxidative stress, suppress inflammatory pathways, and induce apoptosis in cancer cells makes it a promising therapeutic agent [12, 13]. However, despite its high physiological activity, low bioavailability due to water solubility limits the biological utilization of AST [14]. To compensate for this disadvantage, hot melt extrusion (HME) technology was applied.

HME is effective in improving the solubility and bioavailability of poorly soluble drugs such as Biopharmaceutical Classification System (BCS) Class II and Class IV drugs that do not dissolve well in water [15]. The HME process converts crystalline drugs into amorphous forms, thereby enhancing their solubility, dissolution rate, and bioavailability [16]. During HME processing, drugs and polymers are mixed at high temperatures and pressures, dispersing the drug within the polymer matrix [17]. This approach maintains the amorphous state of drugs while enabling controlled drug release [18]. In our previous study, we confirmed that applying HME processing to *H. pluvialis* increased the content of AST and bioactive components [19].

An effective DDS should not only improve drug solubility and bioavailability but also enable precise targeting of cancer cells while minimizing off-target effects. Carbon nanotubes (CNTs), including single-walled carbon nanotubes (SWCNTs), double-walled carbon nanotubes (DWCNTs), and multiwalled carbon nanotubes (MWCNTs), have been widely studied as nanocarriers due to their high surface-to-volume ratio, structural stability, and thermal conductivity [20]. MWCNTs have a completely organic framework made up of carbon atoms, with covalently bonded sp^2^ carbon atoms arranged in a hexagonal honeycomb structure (graphene sheets). These graphene layers are rolled up in multiple layers to form a cylindrical shape to form MWCNTs. The tubular structure of MWCNTs enhances drug loading efficiency and prevents NP aggregation, further improving the stability of the DDS [21]. Our previous study demonstrated that loading AgNPs onto MWCNTs significantly enhanced their physiological activities [22].

CNTs can be internalized into cells through endosome-mediated uptake or direct transport across the cytoplasmic membrane [23]. These mechanisms enhance drug delivery efficiency by enabling direct cytoplasmic release, increasing therapeutic efficacy [24]. However, MWCNTs exhibit poor water solubility and dispersion, which can lead to intracellular aggregation and cytotoxicity [25]. To address these challenges, surface functionalization has been employed to improve CNT solubility, biocompatibility, and dispersion in biological environments [26].

Targeted drug delivery strategies further enhance the therapeutic efficacy of cancer treatments while reducing systemic toxicity. Folic acid (FA), with the chemical formula C_19_H_19_N_7_O_6_, consists of a pteridine ring, *p*-aminobenzoic acid, and glutamic acid [27]. FA is an essential vitamin B derivative involved in nucleic acid biosynthesis and cell proliferation [28]. The FA receptor (FR) is a membrane glycoprotein responsible for FA uptake via endocytosis, with three identified subtypes: FRα, FRβ, and FRγ. Among these, FRα is highly expressed in various cancer cells, including non-small-cell lung cancer, cervical cancer, ovarian cancer, and breast cancer, while its expression remains low in normal tissues [29, 30]. Due to its selective overexpression in cancerous tissues, FA is widely recognized as a tumor biomarker and has been employed in targeted DDSs [31].

Functionalizing CNTs with FA not only improves solubility and biocompatibility but also enhances targeting specificity for FR-overexpressing cancer cells. In this study, we synthesized FA-functionalized, MWCNT-conjugated ZrO_2_ NPs loaded with AST to develop an effective, targeted DDS with enhanced anticancer activity. By integrating AST, ZrO_2_ NPs, MWCNTs, and FA, we aimed to create a nanoplatform capable of selective drug delivery, improved biocompatibility, and enhanced therapeutic efficacy against cancer cells.

## 2. Materials and Methods

### 2.1. Materials

MWCNTs were purchased from the Tokyo Chemical Industry (TCI [Tokyo, Japan]). Zirconium chloride (ZrCl_2_), FA, dicyclohexylcarbodiimide (DCC), N-hydroxysuccinimide (NHS), and 1-ethyl-3-(3-dimethylaminopropyl)carbodiimide (EDC) were bought from Sigma-Aldrich (St. Louis, USA). HaCaT and MDA-MB-231 cells were purchased from the Korea Cell Line Bank (Seoul, Korea). Water-soluble tetrazolium (WST)-1 assay kit (EZ-CyTox, Daeil Lab Service), fetal bovine serum (FBS), penicillin and streptomycin (P/S), Roswell Park Memorial Institute (RPMI), and Dulbecco's modified Eagle's medium (DMEM) were procured from Thermo Fisher Scientific (Massachusetts, USA). Propidium iodide (PI), rhodamine 123 (Rh123), acridine orange (AO), and ethyl bromide (EB) were bought from Sigma-Aldrich (St. Louis, USA). Annexin V-FITC and PI were bought from Invitrogen (Waltham, Massachusetts, USA). Defibrinated sheep blood was bought from Kisanbio (Seoul, Korea).

### 2.2. Synthesis of AZ, AZM, and FAZM

**Scheme 1:**
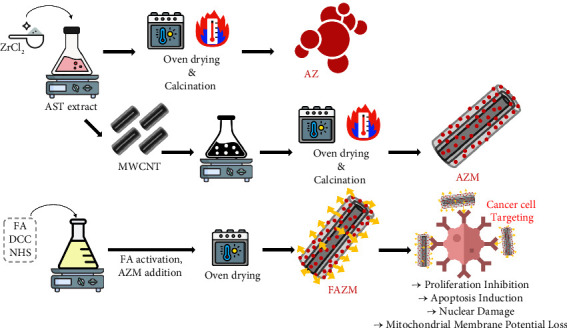
Synthetic process and applications of AZ, AZM, and FAZM.

First, 3 g of *H. pluvialis* powder containing**AST**was added to 100 mL of distilled water and extracted for 1 h using probe sonication (2 s on, 2 s off, amplitude 80%); the composition of AST rich extract is shown in Table 1. For AZ synthesis, 20 mM ZrCl_2_ was added to 100 mL of AST extract and stirred at 80°C for 3 h. The synthesized material was washed three times, dried, and calcined at 600°C to obtain AZ. For AZM, 0.5 g of MWCNTs was added to the unwashed state of AZ, sonicated for 20 min, and stirred for 2 h. The subsequent processes were performed in the same manner as for AZ. Before synthesizing FAZM, 0.5 mM FA was dissolved in 10 mL of DMSO, 0.5 mM EDC and NHS were added, and the mixture was stirred for 3 h to activate FA. Then, 50 mg of AZM was added, stirred at 50°C for 18 h, and washed three times with distilled water. The compositions of AZ, AZM, and FAZM are shown in Table 2, and the synthesis process is illustrated in Scheme 1.

### 2.3. Characterization of AZ, AZM, and FAZM

To observe physical properties, AZ, AZM, and FAZM were analyzed using transmission electron microscopy (TEM) equipped with energy-dispersive spectroscopy (EDS [JEM-2100F, JEOL, Tokyo, Japan]). Selected area electron diffraction (SAED [JEM-2100F, JEOL, Tokyo, Japan]) analysis was performed to confirm the crystallinity of samples. Particle size and polydispersity index (PDI) were measured using dynamic light scattering (DLS) (Zetasizer Nano ZS, Malvern Instruments, Malvern, UK). Zeta potential was measured by Electrophoretic light scattering (ELS) (Zetasizer Nano ZS, Malvern Instruments, Malvern, UK). The chemical compositions were analyzed with a Fourier transform infrared spectrometer (Nicolet iN10, Thermo Fisher Scientific, Waltham, MA, USA) in the range of 400–4000 cm^−1^. Crystallinity was investigated using an X-ray diffractometer (X'Pert-pro MPD, PAN analytical, the Netherlands) in the range of 2*θ* from 5° to 80°.

### 2.4. Antioxidant Activity

The 7.4 mM ABTS solution and 2.4 mM potassium persulfate solution were mixed in a 1:1 ratio and allowed to react in the dark for 16 h. The ABTS solution was diluted with distilled water to adjust the absorbance to 0.7 at 734 nm. Then, 100 μL of the sample and 100 μL of the ABTS solution were added to a 96-well plate and incubated in the dark for 20 min. For the DPPH assay, 100 μL of a 0.1 mM DPPH prepared in methanol solution and 100 μL of the sample were incubated in the dark for 20 min. The absorbance of each plate was measured at 734 and 517 nm for ABTS and DPPH, respectively [21].

### 2.5. Detection of ROS by Fluorescence Staining and Flow Cytometry

To visualize ROS expression, MDA-MB 231 cells were treated with AZ, AZM, and FAZM and cultured for 12 h. Following incubation, the cells were washed with PBS, stained with 2 μM DCFH-DA, and analyzed using a fluorescence microscope.

For quantitative assessment of ROS expression, MDA-MB 231 cells were treated with AZ, AZM, and FAZM and cultured for 12 h. After incubation, the cells were collected by centrifugation and washed with PBS; 1 mL of PBS containing 2 μM DCFH-DA was incubated at 37°C for 20 min. After washing with PBS, the cells were resuspended in PBS and analyzed by flow cytometry at 488 nm [32].

### 2.6. Cytotoxicity

MDA-MB-231 cells were cultured in RPMI, while HaCaT cells were cultured in DMEM added with 10% FBS and 1% P/S at 37°C with 5% CO_2_. Cell viability was assessed using the WST assay. In brief, MDA-MB-231 and HaCaT cells were seeded into a 96-well plate at a density of 2 × 10^4^ cells/well and incubated for 24 h. After incubation, 10 μL of samples at various concentrations were added to each well and incubated for 24 h. Subsequently, 10 μL of WST solution was added to each well, and after incubation for 1 h, the absorbance was measured at 450 nm.

(1) $$\begin{array}{c} \text{Cell viability}\left(\%\right)=\frac{A_{\text{sample}}}{A_{\text{control}}}*100. \end{array}$$

### 2.7. Hemolysis Assay

Blood compatibility was assessed by hemolysis assay according to the previous method [33]. Briefly, red blood cells were prepared by washing defibrinated sheep blood (1 mL) and dispersed in PBS (10 mL). To obtain red blood cells, they were washed three times with PBS. Samples of various concentrations were incubated with red blood cells (200 μL) at 37°C for 1 h A 1% Triton X-100 solution was used as the positive control, while PBS served as the negative control. Following treatment, red blood cells were separated by centrifugation at 2000 rpm for 10 min. The hemolytic activity of the samples was evaluated by measuring the absorbance of the supernatant at 540 nm. The percentage of hemolysis in each sample was calculated relative to the control.

### 2.8. Fluorescence Staining Analysis

MDA-MB-231 cells were seeded in 12-well plates and grown to 90% confluency. They were treated with PBS, AZ, AZM, and FAZM and cultured for 24 h at 37°C with 5% CO_2_. The cells were stained with AO/EB, PI, and RH123 reagents and observed using a fluorescence microscope [34].

### 2.9. Flow Cytometry Analysis

Apoptosis of MDA-MB-231 cells was analyzed using Annexin V-FITC and PI using flow cytometry. The cells were seeded in 6-well plates and cultured at 90% confluency. AZ, AZM, and FAZM were treated at 250 μg/mL and incubated for 24 h. The cells were recovered, washed, and stained with Annexin V and PI [35]. Subsequently, the cells were analyzed in the flow cytometer.

### 2.10. Cell Migration Assay

To evaluate the antiproliferation activity, a cell migration assay was performed. MDA-MB-231 cells were seeded in 12-well plates and incubated. After scratching using a pipette tip, photographs were taken using an optical microscope. After treatment with AZ, AZM, and FAZM and incubation, the cell supernatant was removed, and the medium was replaced. Pictures were taken, and the samples were treated again and incubated. After 48 h of culture, photographs were taken, and the wound closure (%) was calculated using Image J software [36].

### 2.11. Cell Internalization

To evaluate cellular internalization, MDA-MB 231 cells were cultured with the samples for 6 h in a CO_2_ incubator at 37°C. After that, the cells were harvested by trypsinization. The harvested cells were washed with PBS and fixed with formaldehyde (4%) overnight. After that, the cells were washed and dehydrated with increasing ethanol concentrations from 30% to 100%. In addition, dehydrated cells were observed by TEM to visualize cellular internalization. In addition, ICP-OES analysis was used for quantitative analysis of internalization. Briefly, MDA-MB 231 cells were cultured under the same conditions as for TEM analysis, and the cells were washed with PBS, digested with 1 mL of aqua regia (1:3 ratio of HNO_3_ and HCl), and then incubated at 90°C for 3 h. After filtration through a membrane filter with 0.45 μm pore size, the solution was diluted to 10 mL with HNO_3_ (0.2%) and analyzed using ICP-OES (inductively coupled plasma optical emission spectroscopy, Agilent 5900). The quantification of internalized Zr into the cells was calculated using a Zr standard calibration curve [32].

### 2.12. Statistical Analysis

All data were obtained through three replicate experiments, and the results of the analysis are presented as the mean ± standard deviation. Statistical significance was measured through two-way analysis of variance (ANOVA), and a *p* value < 0.05 indicates that the experimental results are statistically significant.

## 3. Results

### 3.1. TEM

**Figure 1:**
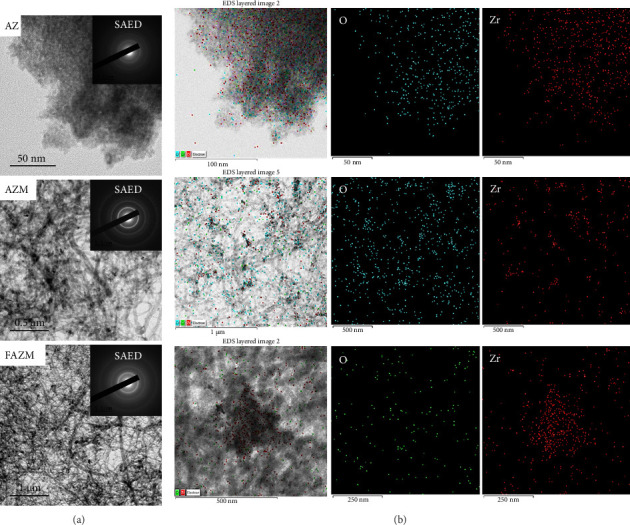
(a) TEM and SAED and (b) EDS images of AZ, AZM, and FAZM.

The morphological characteristics of AZ, AZM, and FAZM were confirmed through TEM analysis (Figure 1). AZ was confirmed to have a highly aggregated and spherical particles less than 50 nm, and AZM was confirmed to be combined with MWCNTs, and particle aggregation improved compared to AZ. FAZM was confirmed to have a similar shape to AZM. SAED analysis can confirm the crystal structure of a specific region of a material [37]. Multiple concentric circles (diffraction rings) were observed in the SAED images of AZ, AZM, and FAZM, which means that the material has a polycrystalline structure. The SAED image of a material with single-crystal characteristics shows a clear spot pattern [38]. The purity of the synthesized AZ, AZM, and FAZM was analyzed by EDS, and the presence of Zr and O elements was confirmed. EDS mapping confirmed that ZrO_2_ NPs were evenly distributed on MWCNTs.

### 3.2. Particle Size, PDI, and Zeta Potential

**Figure 2:**
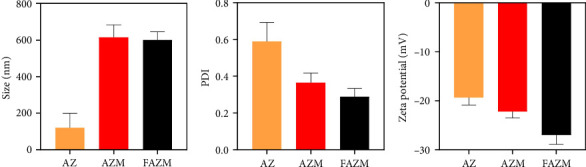
Particle size, PDI, and zeta potential of AZ, AZM, and FAZM.

The particle size, dispersion characteristics, and colloidal stability of AZ, AZM, and FAZM were investigated. The particle size of AZ was confirmed to be 119.33 ± 79.78 d.nm, and the sizes of AZM and FAZM were confirmed to be approximately 500–700 d.nm (Figure 2). The particle size of AZ showed a very large deviation, which is judged to be due to strong particle aggregation, as shown in the TEM analysis. The PDI of AZ was 0.59 ± 0.11, that of AZM was 0.36 ± 0.06, and that of FAZM was 0.28 ± 0.05. The PDI ranges from 0 to 1, where a lower PDI signifies a more uniform particle size distribution and improved dispersion stability [39]. These results suggest that using MWCNTs as a carrier for AZ improves the dispersibility of AZ. The very low PDI of FAZM implies that the aqueous dispersibility of AZM was further improved by functionalizing AZM with FA. The zeta potentials of AZ, AZM, and FAZM were −19.34 ± 1.51 mV, −22.22 ± 1.3 mV, and −27.05 ± 1.83 mV, respectively. The increasing absolute zeta indices indicate improved colloidal stability [40]. These results suggest that stability is improved by combining AZ with MWCNTs and functionalizing it with FA.

### 3.3. X-ray Diffraction (XRD)

**Figure 3:**
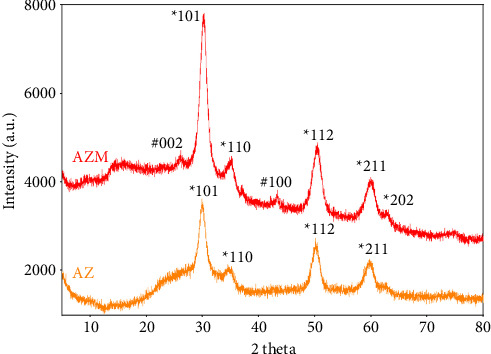
XRD pattern of AZ and AZM. #: Carbon, ∗: ZrO_2_.

The crystal properties of AZ and AZM were investigated at the 2 theta angle (Figure 3). Peaks were identified at 2*θ* = 30.2, 35.0, 50.4, 60.0, and 62.7°, which are attributed to the diffraction of the ∗101, ∗110, ∗200, ∗211, and ∗202 crystal planes of cubic ZrO_2_ (calcination at 600°C), respectively [41]. Crystal planes #002 and #100 represent the characteristics of carbon [42], and these two peaks appeared with relatively weak intensities. This suggests that MWCNTs are surrounded by ZrO_2_ NPs so that crystallinity may be partially observed. This is consistent with the TEM results, which indirectly confirm that ZrO_2_ NPs are uniformly distributed on the surface of MWCNTs and that a composite is formed. XRD analysis suggests the successful bonding of ZrO_2_ NPs and MWCNTs. The crystallinity of AZ and AZM was confirmed to be affected by the calcination temperature. The AZ calcined at 200°C and 400°C did not show any crystal planes corresponding to ZrO_2_, and AZM did not show any crystal peaks except for #002 and #100 of carbon (Figure S1).

### 3.4. Fourier Transform Infrared Spectroscopy (FT-IR)

**Figure 4:**
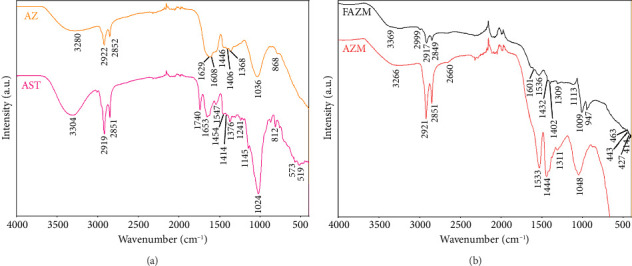
FT-IR spectra of AST, AZ, AZM, and FAZM.

FT-IR analysis was performed to evaluate the functional group changes of each sample and the bond formation between materials (Figure 4). In the peaks of AST, 2919 and 2851 cm^−1^ are signals of C–H bonds, which can confirm the presence of organic compounds [43]. In AZ, these two peaks shifted slightly, and the peak intensities decreased, suggesting that AST was used as a reducing agent for Zr ions, demonstrating the successful synthesis of AZ. The peaks at 1740 cm^−1^ correspond to the C=O (carbonyl) group, suggesting the presence of carboxyl or oxygen-containing functional groups in AST. The peak at 1653 cm^−1^, which represents the amide I vibration band in AST, was confirmed to shift to 1608 cm^−1^ in AZ. This position change is mainly related to the C=O stretching vibration [44]. AST can bind to metal ions to change the electron density of the C=O bond, which causes the peak to shift to a lower frequency. The peaks at 2921, 2851, and 1048 cm^−1^ of AZM are confirmed to be the same peaks as those identified in AST and AZ, suggesting the successful synthesis of AZ and MWCNTs. The peaks at 1533 and 1444 cm^−1^ represent C=C bonds, which are involved in the graphene structure of MWCNTs [45]. The formation of a peak at 1311 cm^−1^ indicates that a C–O bond (carbonyl group) was formed, indicating that a chemical interaction occurred between MWCNTs and AZ [46]. The spectrum of FA showed characteristic absorption peaks located at 1687, 1601, and 1480 cm^−1^, which correspond to the CO amide stretching vibration of the α-carboxyl group, the NH bending vibration of the CONH group, and the absorption band of the phenyl ring, respectively (Figure S2). The disappearance of the CO amide band in FAZM may be due to the formation of FAZM via amide bonds on the AZM surface [47]. The peak at 2999 cm^−1^ in FAZM corresponds to the aliphatic C-H stretching vibration [48]. The peak at 1037 cm^−1^ identified in FA represents the stretching vibration of C–O (carboxyl group, ether) or C–N (amine group), and the peak at 972 cm^−1^ corresponds to the silanol peak [49, 50]. These peaks shifted to 1009 and 947 cm^−1^ in FAZM, which suggests that the peaks shifted toward low frequency due to electronic rearrangement when FA and AZM bind.

### 3.5. Antioxidant

**Figure 5:**
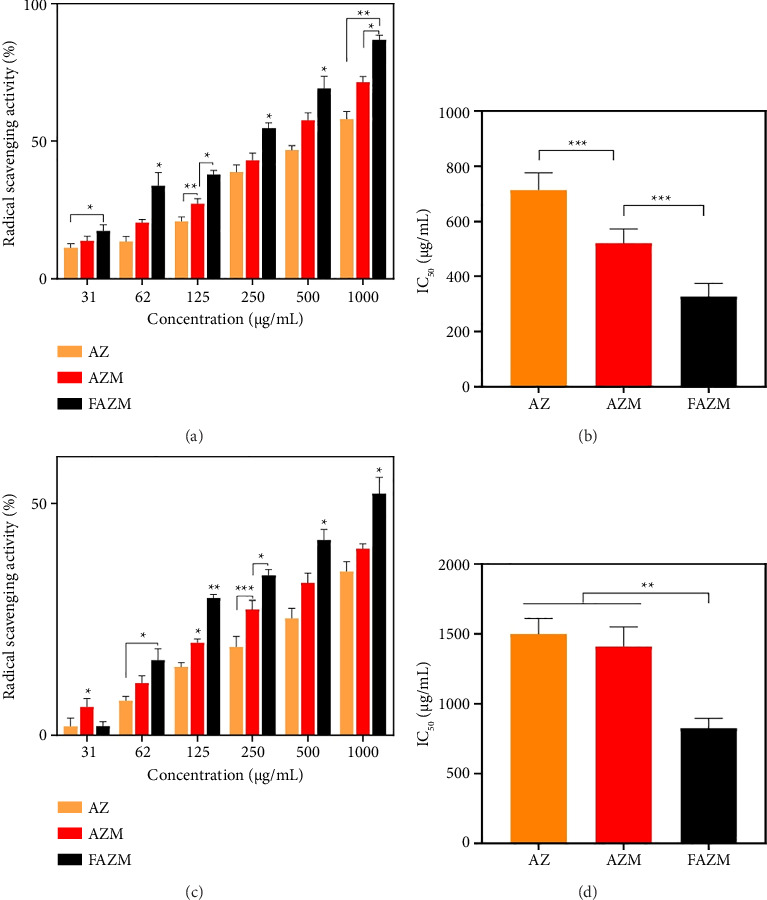
(a) DPPH, (c) ABTS results, and (b, d) IC_50_ values of AZ, AZM, and FAZM. Data are expressed as means ± standard deviation (*n* = 3). ^∗^*p* < 0.1, ^∗∗^*p* < 0.01, and ^∗∗∗^*p* < 0.001.

The antioxidant activities of AZ, AZM, and FAZM were confirmed through DPPH radical scavenging and ABTS radical scavenging assays. The DPPH radical scavenging activities of AZ, AZM, and FAZM were concentration-dependent, and at 1000 μg/mL, the radical scavenging activities of AZ, AZM, and FAZM were 58.06, 71.51%, and 86.99%, respectively, and FAZM showed a significantly increased antioxidant activity (Figure 5(a)). The IC_50_ values of AZ, AZM, and FAZM in DPPH were 713.13 ± 62.63, 520.42 ± 52.08, and 327.70 ± 46.58, respectively (Figure 5(b)). This was a similar trend in ABTS, and FAZM showed the highest antioxidant activity at all concentrations except 31 μg/mL (Figure 5(c)). The IC_50_ values of AZ, AZM, and FAZM in ABTS were 1498.28 ± 112.36, 1408.87 ± 140.85, and 822.78 ± 71.94, respectively, indicating that FAZM significantly enhanced antioxidant activity. This suggests that using MWCNTs as a carrier for AZ and functionalizing them with FA enhances the antioxidant activity of AZ.

### 3.6. Cytotoxicity

**Figure 6:**
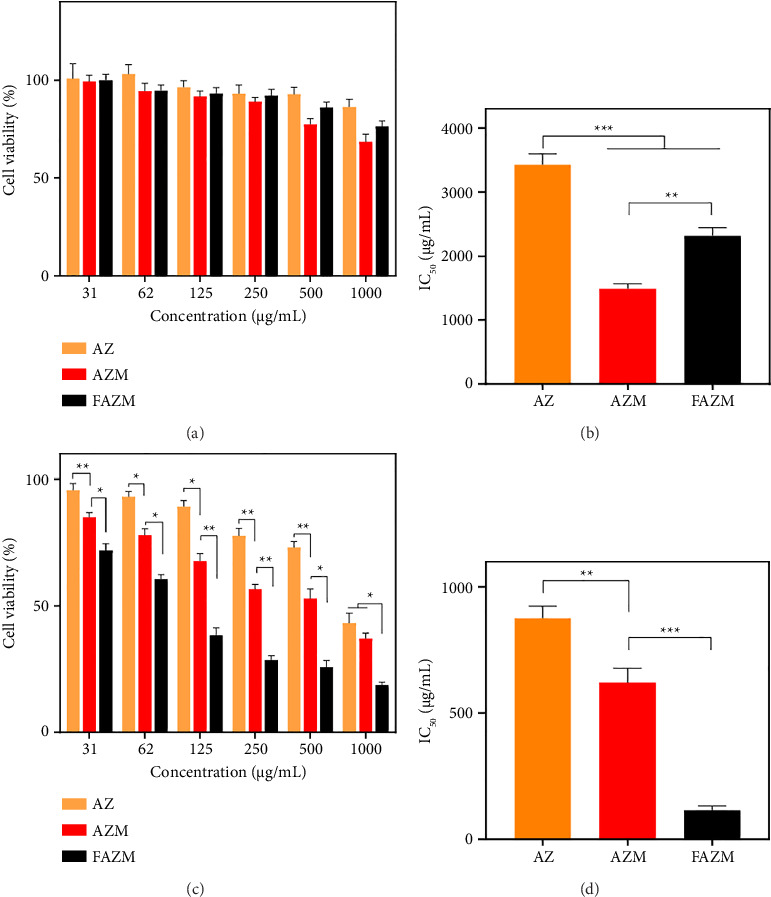
Cytotoxicity in (a) normal human skin cells HaCaT, (c) breast cancer cells MDA-MB-231, and (b, d) IC_50_ values of AZ, AZM, and FAZM. Data are expressed as means ± standard deviation (*n* = 3). ^∗^*p* < 0.1, ^∗∗^*p* < 0.01, and ^∗∗∗^*p* < 0.001.

Cytotoxicity was tested against HaCaT and MDA-MB-231. In the toxicity test on HaCaT, AZ showed no toxicity up to 1000 μg/mL, whereas AZM showed slight toxicity with a cell viability of 77.41% at 500 μg/mL (Figure 6(a)). At 1000 μg/mL, the cell viability of AZM was 68.43%, and that of FAZM was 76.34%, showing that FAZM showed lower cytotoxicity than AZM. The IC_50_ of AZ was 3430.82 μg/mL, indicating very low toxicity. The IC_50_ values of AZM and FAZM were 1488.70 μg/mL and 2314.72 μg/mL, respectively, indicating that FAZM showed improved cytotoxicity than AZM (Figure 6(b)). In the toxicity test on MDA-MB-231, AZ showed low anticancer activity with a survival rate of more than 80% up to 125 μg/mL (Figure 6(c)). AZM and FAZM showed enhanced anticancer activity with survival rates of 67.58% and 38.35%, respectively, at 125 μg/mL. The IC_50_ values of AZ, AZM, and FAZM were confirmed to be 877.31, 620.92, and 115.84 μg/mL, respectively, indicating that FAZM showed approximately 5 times higher anticancer activity than AZM (Figure 6(d)). This indicates that the cytotoxicity of AZ against cancer cells was significantly enhanced through the incorporation of MWCNTs as a delivery vehicle and functionalization with FA.

### 3.7. Hemolysis

**Figure 7:**
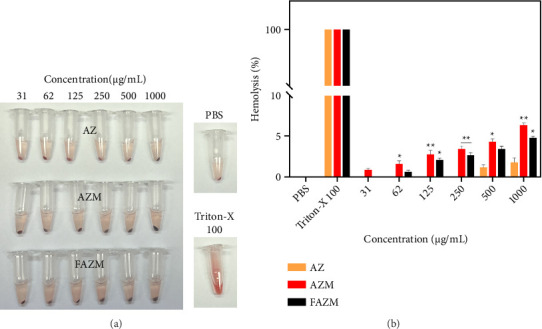
(a) Visualization of hemolysis properties and (b) hemolysis percentage (%). Data are expressed as means ± standard deviation (*n* = 3). ^∗^*p* < 0.1, ^∗∗^*p* < 0.01, and ^∗∗∗^*p* < 0.001.

Blood compatibility is a crucial factor for intravenously administered materials, as it ensures their safe transport through the bloodstream to different tissues. This property is closely linked to hemolysis, which occurs when red blood cells are damaged, leading to the release of hemoglobin into the plasma [51]. The hemolytic effects of AZ, AZM, and FAZM were evaluated on red blood cells (Figure 7). Results show that no significant toxicity (< 5%) was observed up to 500 μg/mL in all samples. However, at 1000 μg/mL, AZM showed some hemolysis (6.34%). Hemolysis exceeding 5% indicates damage to red blood cells due to the material [52].

### 3.8. Detection of ROS by Fluorescence Staining and Flow Cytometry

**Figure 8:**
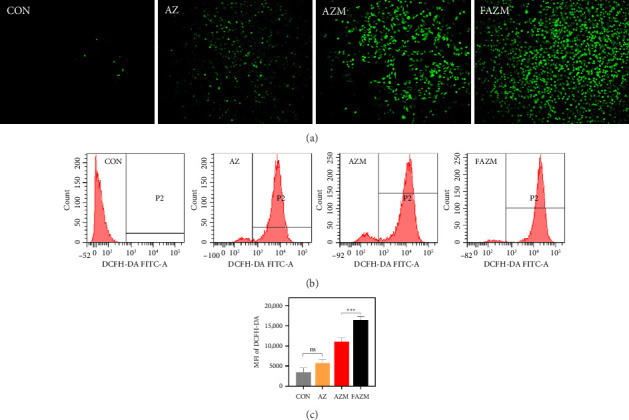
Measurement of ROS expression in MDA-MB-231 using DCFH-DA fluorescence staining analysis (a) and flow cytometry (b), and (c) statistical analysis of (b).

Figure 8 shows the results of visualization and quantitative analysis of intracellular ROS production in MDA-MB-231 breast cancer cell line using DCFH-DA. Green fluorescence indicates intracellular ROS expression, which was barely detected in CON. When AZ was treated, the fluorescence increased slightly, but not significantly. On the other hand, AZM showed very strong fluorescence, and FAZM showed significantly stronger fluorescence intensity than AZM, indicating the highest ROS production.

Quantitative analysis of ROS-induced fluorescence intensity via flow cytometry revealed that FAZM showed the strongest fluorescence intensity. The histogram distribution of the P2 gating region shifted most prominently from left to right in the FAZM-treated group. This strongly suggests that AZM and FAZM induced ROS production more strongly in MDA-MB-231 cells compared to AZ alone. This enhanced ROS generation may be attributed to the induction of oxidative stress through the generation of free radicals such as ROS or reactive nitrogen species (RNS), which is a known mechanism of MWCNT toxicity [53]. Notably, FAZM appears to maximize ROS production by increasing AZM's cellular targeting ability and enhancing its intracellular penetration efficiency. This finding suggests that FAZM possesses significant anticancer or apoptosis-inducing effects, supporting its role as a potent platform to induce ROS-mediated apoptosis.

### 3.9. Fluorescence Staining Analysis

**Figure 9:**
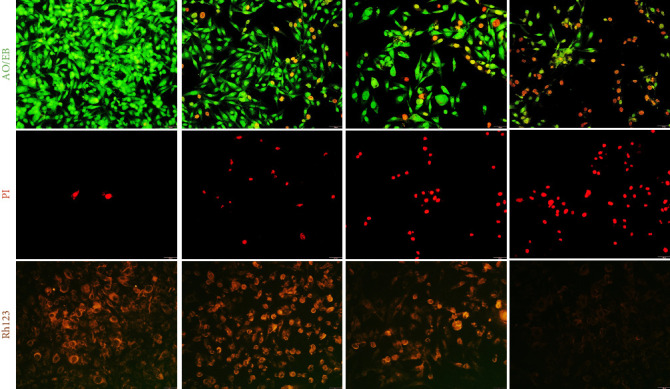
Fluorescence staining analysis results of (a) control, (b) AZ, (c) AZM, and (d) FAZM in MDA-MB-231 cells. The concentration of the samples was 250 μg/mL (scale bar, 50 μm).

AO/EB is a staining agent utilized for assessing cell viability, where live cells emit a green fluorescence, while damaged or dead cells appear red [54]. The anticancer activity of AZ, AZM, and FAZM was visualized by fluorescence staining analysis (Figure 9). AZ showed a slight decrease in live cells and a slight increase in dead cells compared to the control. AZM showed a greater decrease in live cells than AZ, but the increase in dead cells was not significant. FAZM showed a significant decrease in live cells and a significant increase in dead cells, and the morphology of the stained cells was also significantly damaged. PI stains the nucleus of dead cells, and dead cells appear red. In the control group, there were almost no stained cells, and AZ showed a slight increase in the number of stained cells. AZM showed a similar number of stained cells as AZ, while FAZM showed a very significant number of dead cells. Loss of mitochondrial membrane potential in cancer cells can impair essential bioenergetic and metabolic functions, leading to cell apoptosis [55]. Rh123 staining can visualize the loss of mitochondrial membrane potential in cells, and the greater the loss of membrane potential, the weaker the fluorescence intensity [56]. The fluorescence level of AZ was like that of the control, and a slightly reduced fluorescence level was observed in AZM. FAZM showed almost no fluorescence staining, indicating that most cells had lost mitochondrial membrane potential.

### 3.10. Flow Cytometry Analysis

**Figure 10:**
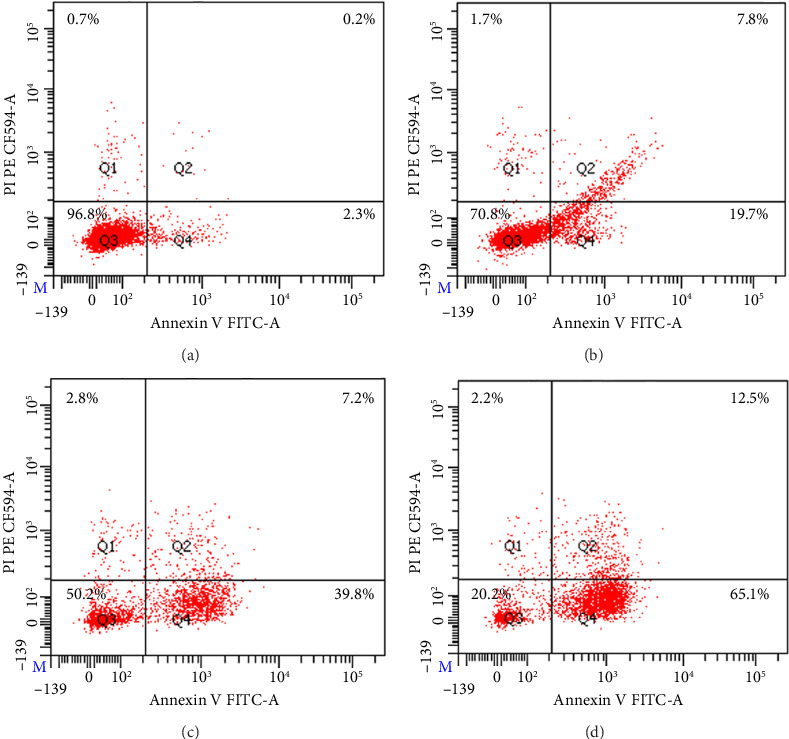
Apoptosis assay for (a) control (PBS), (b) AZ, (c) AZM, and (d) FAZM in MDA-MB-231 cells. The concentration of the samples was 250 μg/mL.

Cells in the apoptosis stage cause abnormalities in the cell plasma membrane, such as asymmetry and changes in permeability, and by measuring these membrane changes, it is possible to determine whether the cell is in apoptosis. Phosphatidyl serine exists inside the cell in healthy cells, but when the cell enters the apoptosis stage, phosphatidyl serine existing inside the cell is exposed to the outside due to membrane changes, and Annexin V specifically binds to the phosphatidylserine molecule [57]. PI penetrates the cell through the damaged cell membrane and binds to DNA, thereby identifying late apoptotic or necrotic cells. In the four quadrants, Q3 indicates cell survival, Q4 indicates early apoptosis, Q2 indicates apoptosis, and Q1 indicates necrosis [58]. In the control, 96.8% of viable cells were detected, confirming that no apoptosis occurred (Figure 10). AZ was detected as 70.8% live cells, 19.7% early apoptotic cells, 7.8% apoptotic cells, and 1.7% necrotic cells. AZM was confirmed as 50.2% live cells, 39.8% early apoptotic cells, 7.2% apoptotic cells, and 2.8% necrotic cells. FAZM was confirmed as 65.1% early apoptotic cells, 12.5% apoptotic cells, and 2.2% necrotic cells, showing a highly significant level of enhanced anticancer activity.

### 3.11. Cell Migration

**Figure 11:**
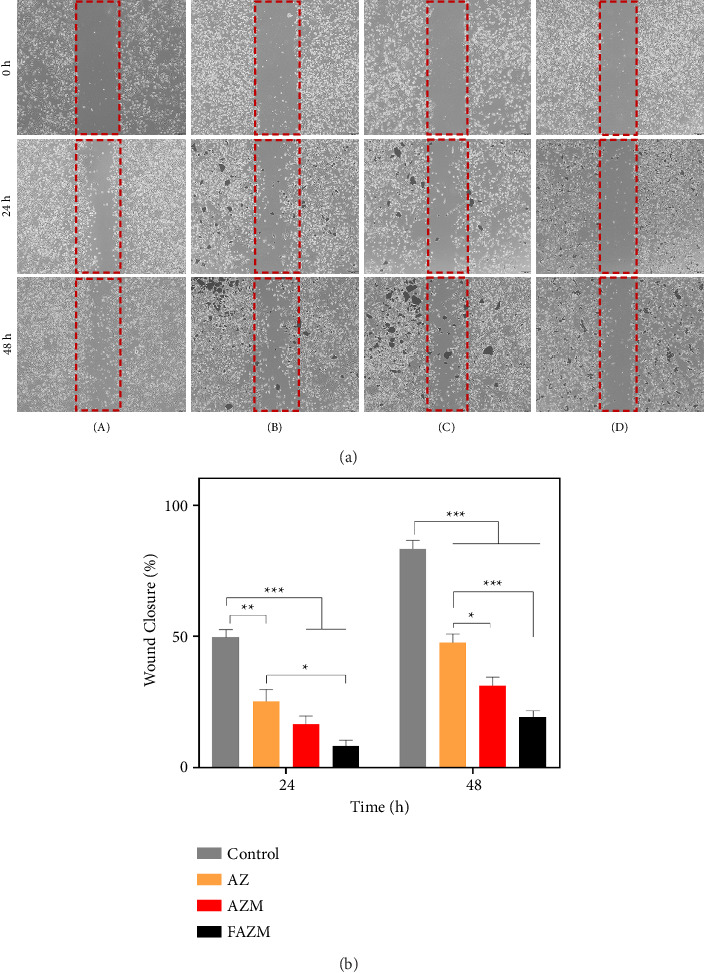
(a) Micrograph of cell migration in MDA-MB-231 treated with (A) control (PBS), (B) AZ, (C) AZM, and (D) FAZM. (b) Quantification of wound closure (%). Data are expressed as means ± standard deviation (*n* = 3). ^∗^*p* < 0.1, ^∗∗^*p* < 0.01, and ^∗∗∗^*p* < 0.001.

Increased proliferation and migration of cancer cells are the basis of tumor invasion, and uncontrolled proliferation can promote metastatic progression [59]. The antiproliferative effects of AZ, AZM, and FAZM were investigated in vitro on MDA-MB-231 cells. The control showed significantly increased wound closure after 24 and 48 h, whereas AZ showed decreased cell proliferation by 25.33% in 24 h and 47.66% in 48 h compared to the control (Figure 11). AZM showed reduced wound closure compared to AZ, which confirmed that using MWCNTs as a carrier for AZ enhanced the cancer cell proliferation inhibition activity. FAZM showed the highest cell proliferation inhibition activity, with wound closure at 24 h at 8.33% and wound closure at 48 h at 19.33%. These results suggest that AST effectively inhibits the proliferation of cancer cells, and MWCNTs can enhance the antiproliferative activity of AZ. In addition, AZM functionalized with FA shows that the target delivery efficiency to cancer cells is further enhanced, which has the potential to maximize the therapeutic effect.

### 3.12. Cellular Internalization

**Figure 12:**
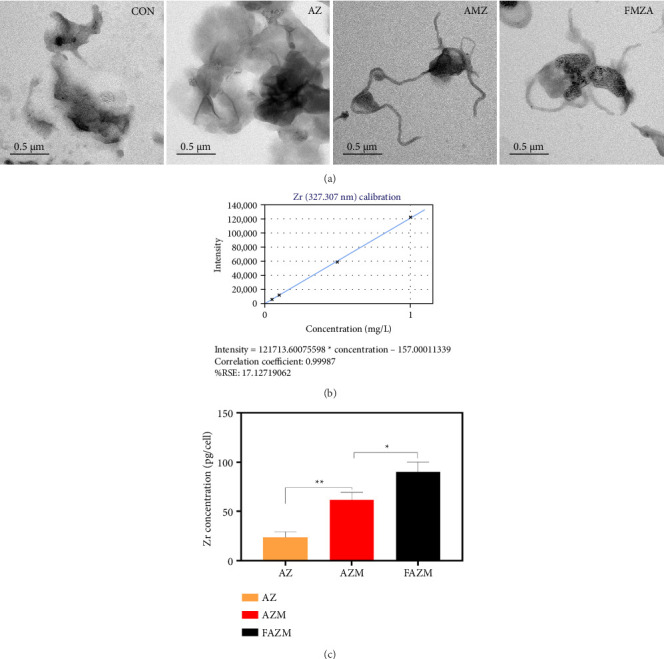
Cellular internalization of AZ, AZM, and FAZM formulations in MDA-MB-231 cells. (a) TEM images showing intracellular localization of the formulations. (b) Zr standard calibration curve for quantitative analysis of intracellular zirconium content using ICP-OES. (c) Quantification of intracellular zirconium content in cells treated with AZ, AZM, and FAZM.

Figure 12 presents the results of TEM analysis and ICP-OES-based quantification of the cellular internalization of AZ, AZM, and FAZM formulations in MDA-MB-231 breast cancer cells. The intracellular penetration and location of each formulation were visually confirmed through TEM images.

In the AZ-treated cells, highly aggregated AZ particles were found to be attached to the cells. This is a result of the strong aggregation of AZ, as in the TEM analysis results of AZ. This aggregation may have occurred due to the lack of surface charge of AZ NPs, low dispersion stability, or nonspecific interaction with the cell membrane, and as a result, they tend to remain on the cell surface rather than efficiently penetrate into the cells [60]. This suggests that there are physicochemical limitations for the AZ-only formulation to be used as an intracellular drug delivery vehicle.

Conversely, in the AZM- and FAZM-treated groups, the MWCNTs and dispersed AZ particles were observed to be wrapped around or internalized by cancer cells. This suggests that the MWCNT-based carrier effectively inhibited the aggregation of AZ and promoted intracellular entry by enhancing cell membrane permeability due to the physical properties of the nanostructure.

The reliability of the quantitative analysis for Zr, a component of the FAZM complex, was confirmed by establishing a linear standard curve (*R* = 0.99987) at the Zr wavelength (327.307 nm). The regression equation and correlation coefficient further support the analytical accuracy. The highest Zr content (90 pg/cell) of FAZM confirmed in the ICP-OES analysis indicates that FAZM was most efficiently internalized into cancer cells, quantitatively demonstrating that the cell permeability and delivery efficiency of the FAZM formulation were superior to those of AZ or AZM. These results suggest that the specific binding to cancer cells was enhanced by functionalizing with FA, which enhanced intracellular uptake [61].

## 4. Discussion

In our previous study, we confirmed that nanocomposites using MWCNTs, AST, and silver nanoparticles (AgNPs) induced toxicity in normal cells. To mitigate this toxicity, ZrO_2_ NPs were employed as a substitute for AgNPs due to their superior biocompatibility. MWCNTs were utilized as carriers for AZ (ZrO_2_ NPs synthesized using AST extract) and were functionalized with FA to enhance the anticancer activity of the nanocomposites while reducing toxicity to normal cells.

The physicochemical properties of AZ, AZM (AZ conjugated with MWCNTs), and FAZM (FA-functionalized AZM) were characterized using TEM, DLS, ELS, XRD, and FT-IR. The characterization data revealed that MWCNTs effectively mitigated the strong aggregation caused by the high surface area of AZ. Additionally, FA functionalization significantly improved the aqueous dispersibility and colloidal stability of AZM. TEM, SAED, and EDS analyses confirmed the successful synthesis of AZ, AZM, and FAZM. The polycrystalline structure observed through SAED analysis enhanced mechanical stability and surface reactivity, making the material useful in applications such as catalysis, biosensing, and nanomedicine. Compared to single-crystal materials, polycrystalline ZrO_2_ NPs exhibit increased defect sites that contribute to their chemical reactivity and biological interactions [62].

Understanding the interaction of synthesized nanocomposites with cells is critical. The physicochemical properties of NPs, including their size, charge, and surface modification, significantly influence their uptake efficiency and internalization pathways. Generally, NP internalization occurs via endocytosis, encompassing clathrin-mediated, caveolae-mediated, and macropinocytosis pathways.

NPs ranging from 10 to 400 nm in size are capable of penetrating the cancer cell membrane, whereas those larger than 500 nm are typically transported into cancer cells via endocytosis [63]. CNTs can enter cells through either cellular internalization pathways or passive diffusion. In the context of cellular internalization, CNTs are initially internalized into endosomes and subsequently translocated to perinuclear lysosomes. This energy-dependent uptake is primarily clathrin-dependent for both SWCNTs and MWCNTs [64, 65].

In antioxidant assays, AZ exhibited low antioxidant activity, while AZM and FAZM demonstrated significantly enhanced antioxidant properties. This improvement is likely due to the reduction of aggregation by MWCNTs, which increased the active molecular surface area of AZ, thereby enhancing electron transfer and stabilizing reactive species [66]. FA exists in dimeric lactam and lactim forms, with the lactim form containing a hydroxyl group on the purine ring that can react with oxidative radicals, further supporting its antioxidant function [67]. FA, a vitamin B complex family member, has been reported to possess high antioxidant activity, which may explain the superior antioxidant capacity of FAZM [68].

Toxicity assays and hemolysis tests conducted on normal human skin cells confirmed that AZ exhibited minimal toxicity and low hemolytic activity, suggesting the biocompatibility of ZrO_2_ NPs and AST. Zirconium has been reported to possess excellent biocompatibility in vivo, making it suitable for biomedical applications such as bone grafts and implants [69]. AST is known for its low toxicity and cytoprotective effects, as it integrates into lipid bilayers and protects membrane phospholipids from oxidative damage [70].

However, the increased toxicity observed in AZM is likely due to the presence of MWCNTs. Previous studies have demonstrated that MWCNTs can induce cytotoxicity in normal mouse fibroblast cells (L929) [22]. The physicochemical properties of MWCNTs, including length, diameter, shape, surface area, and surface chemistry, can contribute to cell membrane damage and cytotoxic effects [71]. One approach to minimizing the cytotoxicity of MWCNTs is the targeted delivery of therapeutic agents, which ensures selective interaction with cancer cells while sparing normal cells [72]. The significant difference in IC_50_ values between AZM and FAZM in HaCaT cells suggests that FA functionalization plays a critical role in reducing normal cell toxicity.

AO/EB staining results revealed a high level of apoptosis in cancer cells treated with FAZM, indicating that FAZM enhances the anticancer activity of AZ through MWCNT conjugation and FA functionalization. FA conjugation is known to facilitate targeted drug delivery by binding to FRs, which are overexpressed in many cancer cells. FRs are 38-kDa glycosylphosphatidylinositol-anchored proteins that are overexpressed only in various tumor cells, including lung, ovarian, brain, head and neck, and breast cancers, while they are found at low levels in healthy cells [73]. This receptor-mediated interaction promotes intracellular uptake, leading to increased drug accumulation and enhanced apoptosis, as observed in fluorescence staining [74].

PI selectively stains the nucleus of late apoptotic or necrotic cells because it cannot penetrate intact cell membranes [75]. PI staining results further confirmed that FAZM treatment significantly increased the number of PI-positive cells, indicating a transition to apoptosis or necrosis. The increased oxidative stress generated by FAZM may contribute to this cytotoxic effect, as metal NPs are known to induce cancer cell death via ROS generation [29]. FA-mediated targeting enhances the intracellular accumulation of AZM in MDA-MB-231 cells, thereby amplifying its apoptotic effect. The relatively lower PI staining in AZM-treated cells suggests that while MWCNTs improve AZ delivery, they do not induce apoptosis as effectively as FAZM.

Mitochondrial membrane potential loss is a hallmark of apoptosis, as it disrupts ATP production and triggers the release of pro-apoptotic factors, such as cytochrome c, thereby activating the caspase-dependent apoptotic pathway [55]. Rh123 staining results suggest that FAZM induces mitochondrial dysfunction, leading to extensive apoptosis. Previous studies have demonstrated that FA-functionalized NPs enhance anticancer activity by increasing cytochrome c expression in cancer cells [76].

Annexin V/PI staining further confirmed the apoptosis-inducing effects of AZ, with apoptosis levels significantly enhanced in AZM-treated cells and reaching the highest levels in FAZM-treated cells. Cancer progression is closely related to ROS accumulation and oxidative stress, which affect DNA damage, tumor metastasis, and drug resistance. AST has been shown to be involved in ROS production, reduce oxidative stress in normal cells, induce cancer cell apoptosis, and inhibit cancer cell proliferation [77]. Additionally, MWCNTs facilitate efficient drug loading, improve drug stability, and enhance targeted delivery to cancer cells, thereby increasing therapeutic efficacy. MWCNTs can also modulate immune responses by interacting with immune cells, potentially inhibiting cancer cell growth [78]. FAZM exhibited the highest percentage of apoptotic cells (77.6%), confirming its superior anticancer efficacy, likely due to FA-mediated targeting of FRs in cancer cells.

Cancer cell migration and invasion are closely linked to ROS signaling [79]. AST, as a potent antioxidant, can regulate ROS production in cancer cells and inhibit proliferation by modulating proliferating cell nuclear antigen and cyclin D1 expression [80]. Malignant tumors metastasize by detaching from the primary tumor, degrading the extracellular matrix (ECM), and invading surrounding tissues [81]. Matrix metalloproteinases (MMPs), particularly MMP-2 and MMP-9, are key regulators of ECM degradation and tumor invasion. AST has been reported to downregulate the expression of MMP-2 and MMP-9, thereby inhibiting tumor cell migration and invasion [82].

ZrO_2_ NPs exhibit high biocompatibility and can penetrate cells via endocytosis, leading to intracellular stress and membrane damage [83]. MWCNTs, due to their high surface area and tubular structure, facilitate high-dose drug loading, improving drug delivery efficiency [84]. Additionally, MWCNTs can disrupt mitochondrial respiration and cytoskeletal assembly, thereby suppressing cancer cell migration and invasion [85]. The significant inhibition of cancer cell migration observed in FAZM-treated cells suggests that FA-mediated targeting enhances the intracellular delivery of AZM, thereby exerting superior antimigratory effects. Previous studies have shown that FA inhibits colon cancer cell proliferation via the FRα/cSrc/ERK1/2/NFκB/p53 pathway and suppresses COLO-205 tumor growth in vivo [86].

One of the primary objectives of cancer treatment is to maximize therapeutic efficacy while minimizing toxicity to normal tissues. In this study, FAZM were developed as a targeted nanoplatform for cancer therapy. FAZM exhibited high antioxidant and anticancer activities while demonstrating reduced toxicity in normal cells compared to AZM. MWCNTs have shown promising potential as nanocarriers, yet concerns persist regarding their poor biodegradability. This characteristic could lead to chronic inflammation, cellular damage, or tissue toxicity with long-term accumulation. Therefore, future studies should focus on improving the biocompatibility and biodegradability of MWCNT-based nanocomposites. It is also crucial to comprehensively evaluate their pharmacokinetics, biodistribution, therapeutic efficacy, and long-term safety using appropriate in vivo models.

## 5. Conclusion

In this study, a cancer cell–targeting drug delivery complex was developed using MWCNTs as a carrier for AZ, functionalized with FA. The conjugation of AZ with MWCNTs and subsequent FA functionalization enhanced its anticancer activity by improving aqueous dispersibility and stability. FAZM exhibited significantly higher antioxidant activity compared to AZ and AZM. In the toxicity test on HaCaT cells, FAZM demonstrated lower cytotoxicity than AZM, confirming that FA functionalization can help reduce toxicity in normal cells. Similarly, in the hemolysis assay, FAZM showed improved biocompatibility compared to AZM. In MDA-MB-231 cells, AZ, AZM, and FAZM exhibited increasing cytotoxicity, nuclear damage-inducing ability, mitochondrial membrane potential loss, apoptosis-inducing ability, and antiproliferative activity in that order, with FAZM showing the highest anticancer activity at a statistically significant level. These results suggest that FAZM has strong potential as an anticancer agent.

## Figures and Tables

**Table 1:** Preparation of AST rich extract by HME processing.

Formulations	
*Haematococcus pluvialis*	70
Lecithin	2.5
Vitamin C	1.5
Vitamin E	1
HPMC	20
Ascorbyl palmitate	5
Total	100 (%)

*Note:* Astaxanthin (AST) and hydroxypropyl methylcellulose (HPMC).

Abbreviation: HME, hot melt extrusion.

**Table 2:** Manufacture of AZ, AZM, and FAZM.

	AST	ZrCl_2_	MWCNTs	FA	Calcination
AZ	O	O			O
AZM	O	O	O		O
FAZM	O	O	O	O	

*Note:* ZrCl_2_: Zirconium chloride, MWCNTs: multiwalled carbon nanotubes, AZ: zirconium oxide nanoparticles (ZrO_2_ NPs) synthesized using AST extract, AZM: AZ conjugated to MWCNTs, and FAZM: AZM functionalized with FA.

Abbreviation: FA, folic acid.

## Data Availability

The data that support the findings of this study are available from the corresponding author upon reasonable request.

## Supporting Information

Figure S1: Crystal characteristics (XRD) results according to the calcination temperature of AZ.

Figure S2: FT-IR results of folic acid.
