# Supplementary material for: Folic Acid–Functionalized MWCNT-Conjugated Zirconium Oxide Nanoparticles for Targeted Cancer Cell Delivery of Astaxanthin
Source: Bioinorg Chem Appl. 2025 Oct 12;2025:4077233. doi: 10.1155/bca/4077233 (PMC12535815; doi:10.1155/bca/4077233)
Supplement: Supporting Information — Additional supporting information can be found online in the Supporting Information section. [file 4077233.f1.docx]

**Supplementary data**


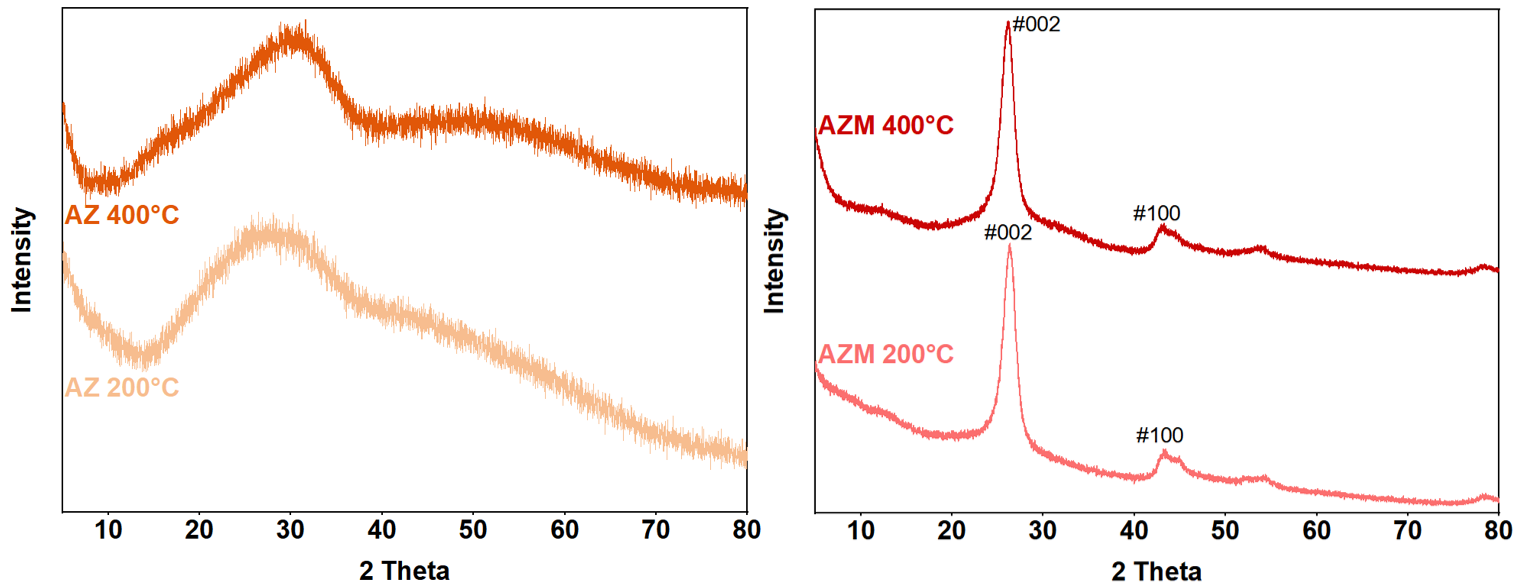


**Figure S1.** XRD patterns of AZ and AZM changing with calcination temperature. #: Carbon.


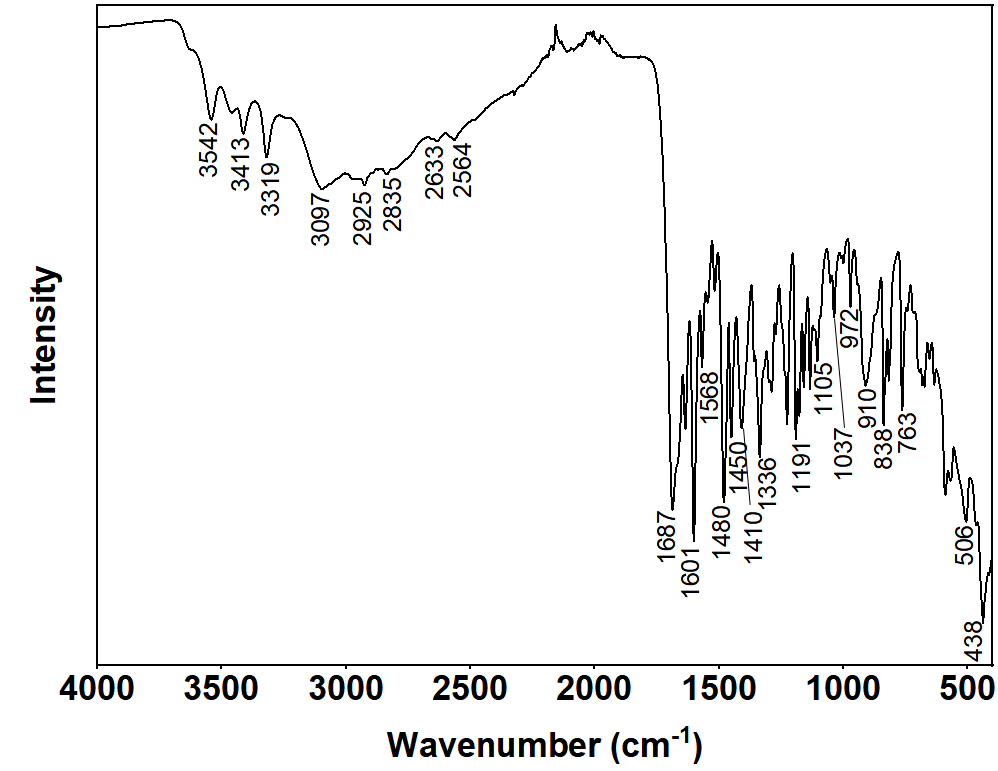


**Figure S2.** FT-IR spectra of Folic acid.
